# Converging mitochondrial MicroRNA Patterns: Ischemic Stroke and Alzheimer's Connections

**DOI:** 10.1002/alz.089510

**Published:** 2025-01-09

**Authors:** Murali Vijayan, Hemachandra Reddy

**Affiliations:** ^1^ Texas Tech University Health Sciences Center, Lubbock, TX USA

## Abstract

**Background:**

The present study delves into the intricate molecular connections between ischemic stroke (IS) and Alzheimer's disease (AD) through an analysis of mitochondrial microRNA (miRNA) patterns. By exploring their shared signatures in the context of IS and AD, our aim is to unravel potential common pathways, understand shared molecular mechanisms, explore diagnostic and therapeutic opportunities, gain a comprehensive understanding of neurodegeneration, and advance the field of biomarker research.

**Method:**

To explore these intriguing questions, mitochondria were isolated from postmortem brains of individuals with IS, AD, and healthy controls (n=10 each). Subsequently, miRNA extraction was carried out using the isolated mitochondria. Following this, a comprehensive analysis of global miRNA profiles in mitochondrial fractions was performed for all three groups, utilizing Hiseq 2000 or 2500 Sequencing & Pipeline filter (Illumina Inc). Furthermore, we employed the Ingenuity Pathway Analysis (IPA) software to elucidate the biological processes, cellular components, and molecular pathways associated with potential mitochondrial miRNAs.

**Result:**

Our results unveil distinct transcriptomic signatures in healthy control, IS, and AD groups. Particularly noteworthy is the identification of significant common mitochondrial miRNA alterations in both IS and AD. Furthermore, the application of IPA to mitochondrial miRNAs reveals their involvement in key signaling pathways, including calcium, Ras, P13K‐Akt, MAPK, and axon guidance. All of these pathways play central roles in the pathogenesis of both ischemic stroke and Alzheimer's disease.

**Conclusion:**

Our study is the first to delineate common mitochondrial miRNA signatures in the states of IS and AD. Furthermore, this comprehensive miRNA‐seq analysis reveals the interconnectedness of these two prevalent neurological disorders at the molecular level, offering valuable insights for future therapeutic interventions and diagnostic strategies.

